# Cyclosporine-A therapy-induced multiple bilateral breast and accessory axillary breast fibroadenomas: a case report

**DOI:** 10.1186/1752-1947-4-267

**Published:** 2010-08-11

**Authors:** Ahmed Darwish, Ayman O Nasr, Lamya A El Hassan, Ahmed H Fahal

**Affiliations:** 1 P.O. Box 102, Department of Surgery, Faculty of Medicine, University of Khartoum, Sudan; 2 Lamya A El Hassan, Department of Pathology, School of Medicine, Ahfaad University for Women, Khartoum - Sudan

## Abstract

**Introduction:**

Breast adenoma is common. However, in the setting of post-transplantation immune suppression it may be expressed differently.

**Case presentation:**

A 35-year-old Sudanese woman, with a history of renal transplantation two and half years prior to presentation, was on a single immune suppression therapy in the form of cyclosporine-A since the transplantation. During a regular follow-up visit, she was noticed to have gingival hypertrophy and bilateral breast and axillary swellings. She underwent successful surgical resection of the bilateral fibroadenomas.

**Conclusions:**

Cyclosporine-A therapy post renal transplantation is associated with an increased incidence of benign breast changes as fibroadenoma. Regular follow-up and appropriate selection of immunosuppressant therapy are essential in the post transplantation management of these patients.

## Introduction

The presence of bilateral breast fibroadenoma is rare. To the best of our knowledge, there are no previous reports of bilateral breast fibroadenoma associated with accessory axillary breast fibroadenoma in the English medical literature. We present the case of a woman with bilateral breast fibroadenoma secondary to cyclosporine-A therapy post renal transplantation.

## Case presentation

A 35-year-old Sudanese woman presented to the Surgical Out-patient Department at Soba University Hospital, Khartoum, Sudan with bilateral breast and axillary swellings. Her condition started one year prior to presentation with a small painless right breast lump slowly increasing in size. It was not considered necessary by our patient to report it during her regular follow-up until seven months later when she noticed multiple small painless nodules on the left breast. She mentioned these lumps to the nephrologist in the following visit. There was no nipple discharge, local skin changes or variation in size with menstruation. One month later she noted bilateral painless axillary swellings that started to increase in size.

Our patient was diagnosed with end-stage renal failure of unidentified etiology three years prior to presentation. She underwent regular hemodialysis sessions before kidney transplantation two years prior to presentation. Her post-operative recovery was uneventful and she was commenced on cyclosporine-A (175 mg/day). She continued to attend regular follow-ups post transplantation. After two years of follow-up, she was found to have gum hypertrophy with no associated pain, bleeding or oral complains.

Her menarche was at the age of 16. She had a regular menstrual cycle and unremarkable adolescence. She had normal breast development, with no past or family history of breast disorders. She never used contraceptive pills.

**Figure 1:**
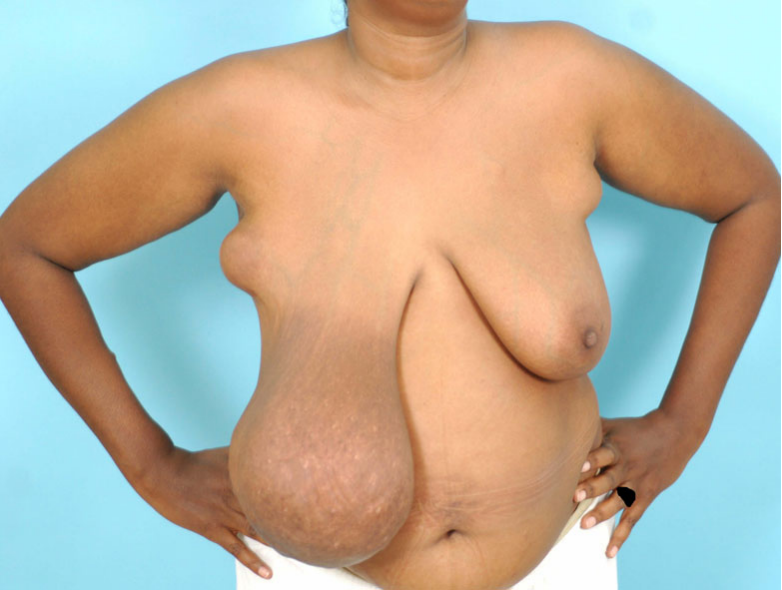
**Photograph showing hugely enlarged right breast and bilateral accessory axillary breast tissue**.

General examination was satisfactory. Oral examination confirmed upper and lower gingival hypertrophy with congestion, good oral hygiene and no ulceration or bleeding spots. There was bilateral accessory axillary breast tissue. Right breast examination revealed a 20 cm mobile firm non-tender mass. Examination of the left breast revealed multiple mobile masses not more than 5 cm in diameter with similar features to the findings in the right breast. A similar mass, 3 cm in diameter, was found within a right accessory axillary breast but no palpable axillary lymph nodes. There were no associated nipple or skin changes. Both accessory axillary breasts tissue contained multiple small masses. The findings of both breasts and axillae were consistent with a clinical diagnosis of fibroadenoma (Figure 1).

Full blood count, liver function test, kidney function test, chest X-ray and echocardiogram showed no abnormalities. Serum prolactin level was also within normal range. Bilateral breast and axillary ultrasound examination supported the clinical findings suggestive of multiple fibroadenomas.

She underwent excision of multiple bilateral breast and axillary fibroadenomas through multiple skin incisions. She had a good post-operative recovery and wound healing. Histopathological examination of all excised masses confirmed the diagnosis of fibroadenomas.

## Discussion

The association between post-renal transplant cyclosporine-A therapy and breast changes is described in the medical literature [1,2]. These changes can be focal or generalized depending on the pathology. Focal changes commonly occur in the form of fibroadenoma, which may be single, multiple, unilateral or bilateral. Low-grade phylloides tumor is another, less common, focal breast change that is associated with cyclosporine-A therapy. A diffuse pattern usually occurs in the form of multiple nodularity, fibrocystic disease or a more uniform generalized pattern with histological features similar to those found in men with gynecomastia [3,4]. Another form of diffuse breast changes is generalized painful breast hypertrophy. This has to be differentiated from inflammatory breast cancer, infiltrative lymphoma and leukemic breast disease by performing a meticulous triple assessment to excluded malignancy in these patients[3,5].

Cyclosporine is also well known to cause gingival hypertrophy as a direct action of the drug or its metabolites on the gingival fibroblasts. A similar mechanism of action may take place in the breast causing breast hypertrophy and fibroadenomata, which could explain the breast changes in our patient. Both cyclosporine-A and feldipine (a calcium antagonist) are reported to induce hypertrophy by causing hyperprolactinemia supported by a raised serum prolactin level post mammoplasty. The fact that this woman had a normal serum prolactin level makes this explanation less likely. Several cyclosporine-A binding proteins are identified in lymphoid and non-lymphoid cells [3,6]. These proteins are claimed to be related to the development of T-cell lymphoma in some patients post renal transplant on cyclosporine-A therapy [1].

Discontinuing the cyclosporine may induce some improvement in early cases, but the breast may not return to pre-transplantation size due to the established breast fibrosis. In some patients where the degree of gynecomastia is small and/or the abnormalities are focal as fibroadenomas, which can be removed surgically, modification of immunosuppressive regimen is usually not necessary. However, in our patient with the presence of multiple fibroadenomas in every breast tissue including the accessory breasts in addition to the gingival hypertrophy, a shift to another immunosuppressive agent (tacrolimus) was found to be a reasonable decision.

## Conclusions

Cyclosporine-A therapy is associated with breast fibroblast proliferation and breast fibroadenoma. Awareness of the association between cyclosporine-A and fibroadenomas should help to achieve the correct diagnosis in patients post transplantation without subjecting them to unnecessary procedures. Early conversion to tacrolimus should be considered in patients who appear to be developing cyclosporine-A induced breast disease and gingival hypertrophy.

## Competing interests

The authors declare that they have no competing interests.

## Authors' contributions

AD was involved in the diagnosis and management of the patient. AON was involved in data collection and writing of the manuscript. LAEH was involved in the tissue diagnosis and literature search necessary for this manuscript. AHF is the senior surgeon supervised diagnosis, management and writing of this manuscript. All authors have read and approved the final manuscript.

## Consent

Written informed consent was obtained from the patient for publication of this case report and any accompanying images. A copy of the written consent is available for review by the Editor-in-Chief of this journal.
